# Coemissive luminescent nanoparticles combining aggregation-induced emission and quenching dyes prepared in continuous flow

**DOI:** 10.1038/s41467-022-33857-x

**Published:** 2022-10-13

**Authors:** Chong Li, Qi Liu, Shengyang Tao

**Affiliations:** 1 State Key Laboratory of Fine Chemicals, Frontier Science Center for Smart Materials, School of Chemical Engineering, Dalian University of Technology, Dalian, 116024 China; 2 Department of Chemistry, School of Chemical Engineering, Dalian University of Technology, Dalian, 116024 Liaoning China

**Keywords:** Light harvesting, Nanoparticle synthesis, Self-assembly

## Abstract

Achieving an ideal light-harvesting system at a low cost remains a challenge. Herein, we report the synthesis of a hybrid dye system based on tetraphenylene (TPE) encapsulated organic dyes in a continuous flow microreactor. The composite dye nanoparticles (NPs) are synthesized based on supramolecular self-assembly to achieve the co-emission of aggregation-induced emission dyes and aggregation-caused quenching dyes (CEAA). Numerical simulations and molecular spectroscopy were used to investigate the synthesis mechanism of the CEAA dyes. Nanoparticles of CEAA dyes provide a platform for efficient cascade Förster resonance energy transfer (FRET). Composite dye nanoparticles of TPE and Nile red (NiR) are synthesized for an ideal light-harvesting system using coumarin 6 (C-6) as an energy intermediate. The light-harvesting system has a considerable red-shift distance (~126 nm), high energy-transfer efficiency (Φ_ET_) of 99.37%, and an antenna effect of 26.23. Finally, the versatility of the preparation method and the diversity of CEAA dyes are demonstrated.

## Abstract (web summary)

Developing efficient light harvesting systems at low cost is a challenge. Here, the authors synthesized coemissive dyes in a continuous flow microreactor featuring a controlled cascade FRET process combining aggregation-induced emission and quenching.

## Introduction

Light is a resource on which human beings depend and is one of the essential energy sources on earth^1,2^. People have a long history of using and researching light. Today optical materials research plays an important role in various fields, such as solar cells^3,4^, flow photochemistry^5,6^, biofluorescent probes^7,8^, artificial photosynthesis^9–11^, and photodetectors^12,13^. Scientists have been keen on tuning the properties of optical materials to achieve the specific needs of light in their respective fields, such as fluorescence lifetime^14,15^, quantum yield^16,17^, excitation means^18–20^, etc. Organic dyes are one of the classical optical materials, but their susceptibility to aggregation-caused quenching (ACQ) effects in aqueous solutions has limited the scope of conventional organic dyes. In 2001, Ben-Zhong Tang proposed the concept of aggregation-induced emission (AIE)^21^, which significantly improved the range of options for aqueous dyes^22^. In addition, studies have been conducted to combine conventional organic dyes or AIE dyes to mimic the photosynthesis of cells for the light-harvesting process^23,24^. In general, an ideal artificial light-harvesting system should have an energy-transfer efficiency close to 100% and an antenna effect greater than 10^25^. At present, there are few reports of ideal light-harvesting nanoparticle systems constructed using commercially available dyes based on a cascade FRET process.

It makes sense to encapsulate organic dyes with nanoparticles to avoid fluorescence loss due to the ACQ effect in the water. The encapsulated NPs can be used in fluorescence imaging^26,27^, light-harvesting^28,29^, optical anti-counterfeiting^30,31^, etc. Here, we controlled the strength of the hydrophobic interaction by modulating the concentration difference between ACQ dyes (C-6, LR305, and NiR) and TPE and thus synthesized a series of ACQ@AIE-type composite dyes (CEAA dyes). The relative molecular masses of TPE, C-6, and NR are on the same order of magnitude, and the mass ratio between the dyes is used uniformly in the text. The preparation of luminescent nanoparticles by precipitation is efficient^32,33^. In order to control the synthesis conditions more precisely, the whole synthesis process was carried out in a 3D printed jet-type microreactor (Supplementary Fig. 1), which has been shown to possess the advantage of enhanced mass transfer during nanoparticle synthesis^30,34^. While synthesizing CEAA dyes, the preparation mechanism and the synthesis conditions of CEAA dyes were investigated by further experiments and finite-element calculations. CEAA NPs can provide an efficient FRET platform. For example, three FRET processes can exist simultaneously in C-6&NiR@TPE NPs. We obtained C-6&NiR@TPE NPs with light-harvesting ability close to the ideal state by adjusting the content of the intermediate C-6. Finally, a series of CEAA dyes with different emission wavelengths were prepared using the classical AIE dyes, such as hexaphenylsilole (HPS) and tetraphenylthiophene (TP) instead of TPE. The universality of the CEAA dye preparation method and the variety of CEAA dyes were further demonstrated.

## Results

### The preparation process of CEAA dyes

**Fig. 1:**
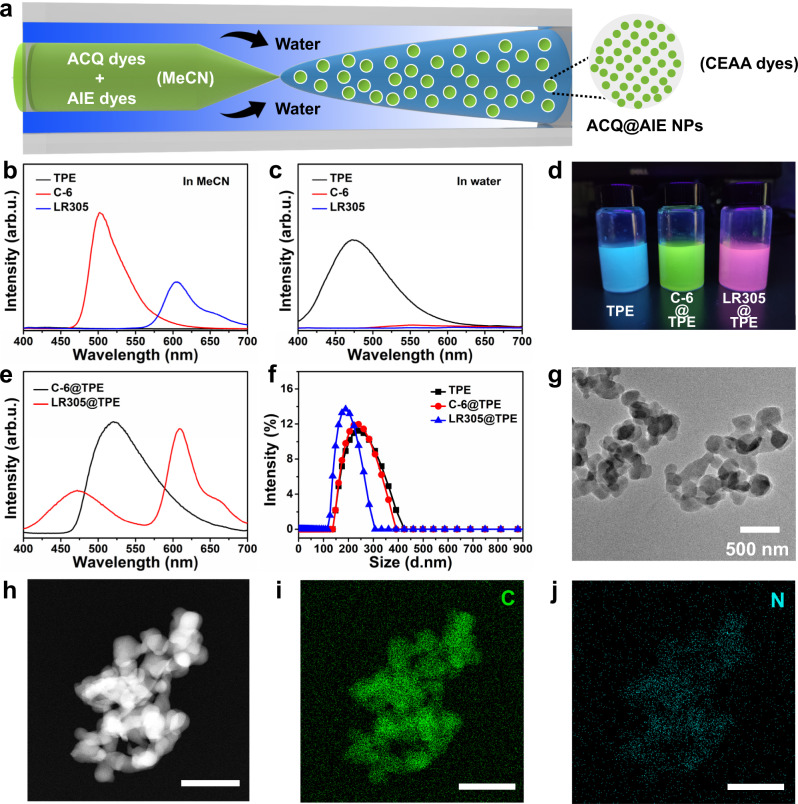
Synthesis and characterization of CEAA dyes. **a** Schematic diagram of the synthesis of CEAA dyes using a continuous flow microreactor. **b** Fluorescence spectra of TPE, C-6, and LR305 in acetonitrile. **c** Fluorescence spectra of TPE, C-6, and LR305 in water. **d** Optical photos of TPE, C-6@TPE, and LR305@TPE in water under 365 nm UV light. **e** Fluorescence spectra of C-6@TPE and LR305@TPE in water. **f** Particle size distribution of TPE, C-6@TPE, and LR305@TPE in water. **g** TEM image of C-6@TPE NPs. **h**–**j** EDS results for elements C and N in C-6@TPE. All bars are 500 nm. The internal and external phase flow rates are 60 and 600 μL min^−1^, respectively. Source data are provided as a Source Data file.

Figure 1a shows a schematic diagram of the preparation of CEAA dyes using a continuous flow microreactor. The inner phase fluid in the capillary tube is acetonitrile (MeCN) solution of precursor (TPE and organic dye), and the outer square fluid channel is filled with antisolvent water. The precursor solution is ejected through the capillary and precipitates in the water to form the CEAA dye. Figure 1b, c shows the fluorescence spectra of TPE, C-6, and LR305 in aqueous and acetonitrile solutions, respectively. TPE does not produce fluorescence emission in acetonitrile solution but undergoes an aggregation-induced emission effect in the water. In contrast, the other two organic dyes undergo the ACQ effect in water and can produce fluorescence emission in acetonitrile solution. Since the concentration of TPE (1 mg mL^−1^) in the precursor was higher than that of the ACQ dye (0.05 mg mL^−1^), two CEAA dyes, C-6@TPE and LR305@TPE, shown in Fig. 1d, were prepared. The ACQ dyes emit fluorescence after being encapsulated in TPE. Figure 1e shows the fluorescence emission spectra of the two CEAA dyes.

The characteristic peak of C-6 in the C-6@TPE dye was more obviously redshifted than that of C-6 in MeCN, meaning that the C-6 had partly aggregated. The character emission of the aggregated TPE at 472 nm was not observed. It may be the energy transfer from TPE to C-6. In LR305@TPE, LR305 maintained the original emission peak position due to its larger molecular volume and unfavored energy level match, which was less prone to multimolecular aggregation and energy transfer. Figure 1f shows the particle size distribution of the TPE, C-6@TPE, and LR305@TPE products, all with an average particle size of approximately 250 nm. The TEM images of C-6@TPE NPs and their energy dispersive X-ray spectroscopy (EDS) results (Supplementary Table 1) are shown in Fig. 1g–j. The uniform distribution of N elements in the C-6@TPE NPs indicated that the C-6 dye was also uniformly encapsulated in the TPE.

### Mechanism of CEAA dye preparation

**Fig. 2:**
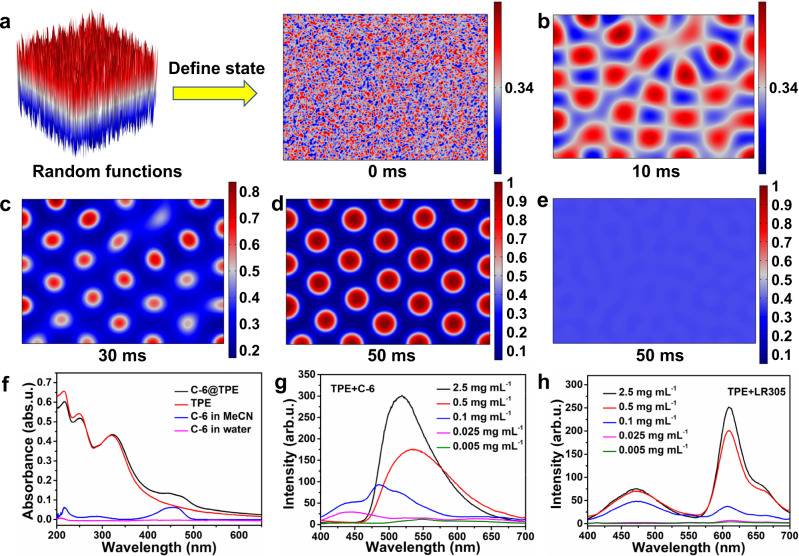
The mechanism of CEAA dyes synthesis. **a** The random function defines the initial state of the phase field diagram of the TPE. **b** Phase field diagram of TPE at 10 ms. **c** Phase field diagram of TPE at 30 ms. **d** Phase field diagram of TPE at 50 ms. **e** Phase field diagram of C-6 at 50 ms. **f** UV–Vis absorption spectra of C-6, TPE, and C-6@TPE dyes. **g** Fluorescence spectra of C-6+TPE obtained with different concentrations of TPE precursors. **h** Fluorescence spectra of LR305 + TPE obtained with varying concentrations of TPE precursors. The geometric size of the simulation area is 2.8 μm × 2 μm. The internal and external phase flow rates are 60 and 600 μL min^−1^, respectively. Source data are provided as a Source Data file.

The key to the preparation of CEAA dyes is the proper concentration difference between the TPE and the organic dye. In antisolvent precipitation, a higher solute concentration makes it easier to precipitate. When a higher concentration of TPE and a lower concentration of C-6 are sprayed into the water simultaneously, the TPE will preferentially precipitate as a solid, and C-6 will aggregate but will not precipitate at the exact moment. According to the principle of “like dissolves like”, C-6 will be encapsulated by TPE instead of remaining in the aqueous phase. This process was simulated by the phase field method based on finite-element analysis. The mixture of water and acetonitrile was considered the solvent phase, and the single solute was the other phase. The initial state of the phase field was first defined using a stochastic function (Fig. 2a), such that the single solute was uniformly distributed in the solution. Figure 2b–d shows the phase field of TPE (1 mg mL^−1^) at different times, where the TPE in solution aggregates and precipitates with time. Within 50 ms, TPE would be completely precipitated as nanoparticles of approximately 250 nm, which fit well with the experimental results. Additionally, at 50 ms, C-6 (0.05 mg mL^−1^) did not aggregate to produce nanoparticles (Fig. 2e) until 70 ms, when it started to aggregate and precipitate (Supplementary Fig. 2). Therefore, C-6 would be encapsulated by TPE NPs to form C-6@TPE dyes. The difference between the simulated results and the real precipitation time of the TPE is not significant (Supplementary Fig. 3, 4).

Figure 2f shows the UV–Vis absorption spectra of each dye. Due to aggregation and precipitation in an aqueous solution, the spatial distribution of C-6 in the solution was low, so the characteristic absorption peak of C-6 (in water) was not detected. When C-6 was uniformly encapsulated in the higher concentration of TPE NPs, which disperse well in the water, the C-6@TPE had the characteristic absorption peaks of TPE and C-6 (in MeCN), and no new absorption peak appeared. In addition, the IR spectra showed no significant changes in the characteristic peaks of TPE after C-6 doping (Supplementary Fig. 5). TPE and C-6 should be physically mixed. These results indicated that C-6 in the C-6@TPE did not precipitate as nanoparticles alone, consistent with the simulation results. The TEM results also did not reveal significant C-6 aggregation in C-6@TPE (Supplementary Fig. 6). Figure 2g shows the fluorescence spectra of the C-6@TPE product when only the concentration of TPE in the precursor was changed, and the flow rate ratio of the inner and outer phases was kept at 1:10. When the concentration of TPE decreased from 2.5 mg mL^−1^ to 0.1 mg mL^−1^, the fluorescence of C-6 decreased, and the emission of TPE appeared. When the concentration of TPE changed from 0.1 mg mL^−1^ to 0.025 mg mL^−1^, a blue-shifted emission peak of TPE first appeared at 440 nm, and then all the emission peaks became very weak. The peak at 440 nm indicated that the TPE was not fully aggregated (Supplementary Fig. 7). Moreover, this blue-shifted peak did not appear for pure TPE at the corresponding concentration (Supplementary Fig. 8). The reason is that the TPE concentration (0.025 mg mL^−1^) was lower than C-6 (0.05 mg mL^−1^). Therefore, C-6 precipitated first (Supplementary Fig. 9) and encapsulated TPE to form TPE@C-6 dye, resulting in incomplete aggregation of TPE. As an ACQ dye, the fluorescence emission peak of the aggregated C-6 largely disappeared. Therefore, the luminescence characteristics of the products were adjusted by regulating the content difference between the two dyes. Figure 2h shows a similar conclusion that the ratio of LR305 to the TPE characteristic peak gradually decreased when the TPE concentration in the precursor steadily reduced, which meant that the ability of TPE to encapsulate LR305 gradually became weaker.

### CEAA dyes provide a platform for efficient FRET

**Fig. 3:**
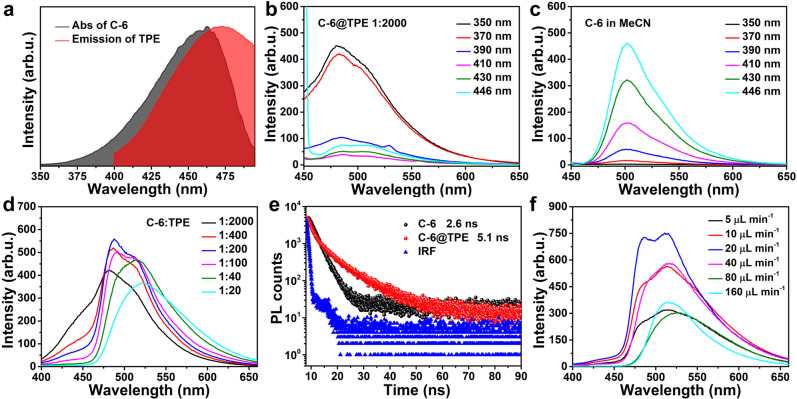
FRET process between TPE and C-6. **a** Emission spectrum of TPE with the absorption spectrum of C-6. **b** Fluorescence spectra of the products at different excitation wavelengths for a precursor C-6 to TPE mass ratio of 1:2000. **c** Fluorescence spectra of the precursors at different excitation wavelengths for a mass ratio of C-6 to TPE of 1:2000. **d** Fluorescence spectra of C-6@TPE at 350 nm excitation light when different ratios of C-6 to TPE were used in the precursors. **e** The fluorescence lifetime of C-6 (in MeCN) and C-6 in C-6@TPE at a mass ratio of 1:20. IRF (instrument response function). **f** Fluorescence spectra of C-6@TPE prepared with different internal phase flow rates at a mass ratio of 1:20. Source data are provided as a Source Data file.

As shown in Fig. 3a, the emission spectrum of TPE overlaps with the absorption spectrum of C-6 over a large area. The mass ratio of C-6 to TPE in the precursor was adjusted to 1:2000, and C-6 (in MeCN) was barely excitable (Fig. 3c) under shortwave light (e.g., 350 nm). Conversely, the TPE was excited by shortwave light instead of longwave light (Supplementary Fig. 10). The FRET effect allowed the C-6 component in the C-6@TPE to be excited by shortwave light (Fig. 3b, excitation peak position: 482 nm). As a result, the excitation intensity was higher than that using longwave light excitation. By increasing the content of C-6, the emission peak of C-6 was gradually redshifted (Fig. 3d), implying that the energy acceptor of the FRET process is an aggregation of multiple C-6 molecules and the energy gap of the acceptor is reduced (Supplementary Fig. 11, see below)^35^. The solid-state fluorescence data showed the same trend (Supplementary Fig. 12). It indicated that during the CEAA preparation, the C-6 did not precipitate as nanoparticles, but the molecules were still partially aggregated. As shown in Fig. 3e, the fluorescence lifetime of C-6 in C-6@TPE increased to 5.1 ns compared to C-6 (in MeCN, 2.6 ns) after the aggregation of C-6 molecules (Supplementary Table 2). The fluorescence lifetime of the assembled TPE decayed due to the FRET process (Supplementary Fig. 13, Supplementary Table 3). In addition, the flow rate ratio of the inner and outer phases of the microreactor also affects the luminescence properties of the products (Fig. 3f). The external phase flow rate was kept at 600 μL min^−1^ and the internal phase flow rate varied from 5 μL min^−1^ to 160 μL min^−1^. When the internal phase flow rate of the microreactor was low (<40 μL min^−1^), the spectrum of C-6@TPE was blue-shifted. It was because the dye concentration of TPE and C-6 in the microchannel decreased simultaneously, resulting in a lower encapsulation of C-6 in TPE.

**Fig. 4:**
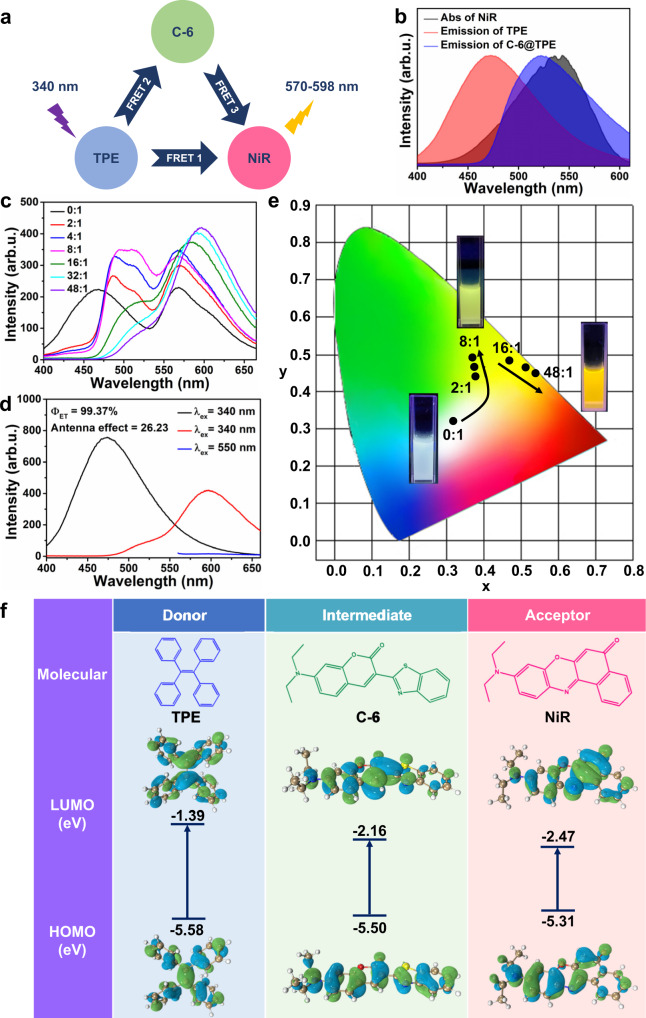
Controlled cascade FRET process for light-harvesting. **a** Schematic diagram of the FRET process in C-6&NiR@TPE dye. **b** Emission spectra of TPE and C-6@TPE with absorption spectra of NiR. **c** Fluorescence spectra and **e** CIE chromatograms of products with different C-6 to NiR mass ratios in precursors when the ratio of TPE to NiR was fixed at 200:1. **d** Fluorescence spectra of TPE and C-6&NiR@TPE used to calculate the energy-transfer efficiency and antenna effect. **f** HOMO and LUMO orbital distributions and calculated orbital energies of TPE, C-6, and NiR molecules. The internal and external phase flow rates are 60 and 600 μL min^−1^, respectively. Source data are provided as a Source Data file.

To evaluate the light-harvesting ability of the CEAA, the classical Nile red (NiR) was selected as the acceptor. As shown in Fig. 4b, the emission spectrum of TPE only partially overlapped with the absorption spectrum of NiR, which implied a limited FRET process between the two. Since the excitation wavelength of the C-6@TPE could be adjusted from 482 nm to 523 nm depending on the content of C-6 (Fig. 3d), C-6 would act as an intermediate responsible for energy transfer from TPE to NiR. Based on this, the C-6&NiR@TPE dye system was prepared. As shown in Fig. 4a, this dye system had a FRET 1 process and a cascade FRET process (FRET 2 + FRET 3). The mass ratio of TPE (0.5 mg mL^−1^) to NiR (0.05 mg mL^−1^) in the precursor was kept at 200:1, and the content of C-6 was gradually increased to regulate the cascade FRET process. As shown in Fig. 4c, the mass ratio of C-6 to NiR in the precursor gradually increased from 0:1 to 48:1. When the C-6 content was in the range of 2:1 to 8:1, the system mainly underwent the FRET 2 process, and FRET 1 and FRET 3 processes existed. When the C-6 content was in the range of 16:1 to 48:1, the emission peak of C-6 was gradually redshifted and entered the absorption spectral region of NiR, which significantly promoted the FRET 3 process. As a result, the emissions of TPE and C-6 were weakened, and the emission of NiR was enhanced.

It was more intuitive from the CIE chromaticity diagram in Fig. 4e that the system change was dominated by FRET 2 when the C-6 content increased from 2:1 to 8:1. When the ratio of the two increased from 16:1 to 48:1, the system change was dominated by FRET 3. The C-6&NiR@TPE system had an energy-transfer efficiency of 99.37%, an antenna efficiency of 26.23, and an ultralong red-shift distance of 126 nm (Fig. 4d). Since the organic dyes are uniformly dispersed and immobilized in the solid CEAA NPs, this will facilitate a stable and efficient FRET process. The fluorescence lifetime of the TPE in this system was significantly reduced and below 0.4 ns (Supplementary Fig. 14 and Supplementary Table 4). In addition, density functional theory (DFT) calculations (Fig. 4f) showed that the lowest unoccupied molecular orbital (LUMO) of TPE was calculated to be −1.39 eV, and the highest occupied molecular orbital (HOMO) was calculated to be only −5.58 eV, with a higher energy gap of 4.19 eV. The lower HOMO energy meant that TPE was more likely to generate electron excitation and was a more desirable energy donor. In comparison, the calculated LUMO value for C-6 was −2.16 eV, while NiR was only −2.47 eV, which meant that they were more prone to electron reception and were more desirable energy acceptors^36^. The proper energy gradient between the three dyes facilitated the efficient antenna effect of the system^25^.

### The universality of CEAA dyes preparation methods

**Fig. 5:**
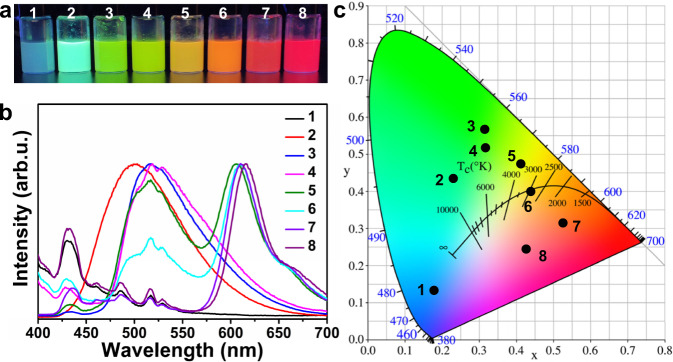
The universality of CEAA dyes preparation. **a** Optical photographs of CEAA with different emission wavelengths under UV light (No. 1–8). **b** Fluorescence spectra and **c** CIE chromatograms of CEAA dyes 1–8. The internal and external phase flow rates are 60 and 600 μL min^−1^, respectively. Source data are provided as a Source Data file.

In addition to TPE, CEAAs with different emission wavelengths were prepared by combining two classical AIE dyes, HPS and TP, with organic dyes (C-6 and LR305). The conditions for the preparation of CEAA are shown in Table 1. Figure 5a shows the optical photographs of CEAA dyes under UV light. Figure 5b, c shows the fluorescence emission spectra and CIE images of the CEAA dyes. The prepared CEAA dye particle size was 200-300 nm (Supplementary Fig. 15). Keeping the appropriate concentration difference between AIE and ACQ dyes, the preparation of CEAA is universal. CEAA products with different emission characteristics can be easily prepared by doping with other dyes to suit the needs of various fields.

**Table 1:** Preparation conditions of CEAA dyes

Entry	Precursor type	Mass ratio
1	TP	/
2	HPS	/
3	HPS + C-6	20:1
4	TP + C-6	20:1
5	HPS + C-6 + LR305	200:9:1
6	TP + C-6 + LR305	200:9:1
7	HPS + LR305	20:1
8	TP + LR305	20:1

## Discussion

We prepared ACQ@AIE-type nanoparticles (CEAA dyes) using a continuous flow microreactor. The concentration difference of different dyes can control the preparation of CEAA, an the CEAA achieves co-emission of ACQ and AIE dyes in the same nanoparticle. CEAA, based on the TPE system, provides an efficient FRET platform. C-6&NiR@TPE dyes have the ultraefficient light-harvesting ability with a high energy-transfer efficiency of 99.37%, an antenna effect of 26.23, and a long red-shift distance of 126 nm. The efficient light-harvesting ability is attributed to (1) the necessary conditions for a stable FRET process since the organic dyes are uniformly dispersed and immobilized in the AIE solid particles; (2) the intermediate C-6 has adjustable emission peak positions, thus achieving an efficient cascade FRET process; and (3) the three selected dyes have gradually decreasing LUMO energy levels and increasing HOMO energy levels, which will also facilitate the FRET process. This investigation guides the preparation of CEAA light-emitting materials. Moreover, CEAA dyes have an efficient light-harvesting ability and are expected to be applied in biomimetic artificial light harvesting.

## Methods

### Materials

All reagents were used as received and were not further purified. N, N′-Bis(2,6-diisopropylphenyl)-1,6,7,12-tetraphenoxyperylene-3,4:9,10-tetracarboxdiimid (LR305, 95%, TCI, CAS Registry Number: 123174-58-3). Coumarin 6 (98%, aladdin). Acetonitrile (AR, aladdin). Nile red (95%, aladdin). Tetraphenylene (98%, aladdin). Tetraphenylthiophene (98%, aladdin). Hexaphenylsilole (98%, macklin).

### Measurements

All fluorescence spectra were acquired by G9800A fluorescence spectrometer. UV-visible absorption spectra were acquired by a TU-1900 spectrophotometer. The fluorescence lifetimes of all the products were acquired by FLS1000 (EPL-300) fluorescence spectrophotometer. All particle size distribution results were collected by Litesizer 500 (Anton Paar). TEM images were measured by JEM-F200. IR data were measured by Nicolet 6700. All surface tension coefficients were determined by OCA50.

### Precursor solution

If not otherwise specified, the solution to configure the precursor is as follows: TPE (MeCN, 2 mg mL^−1^); TP (MeCN, 2 mg mL^−1^); HPS (MeCN, 2 mg mL^−1^); LR305 (MeCN, 0.1 mg mL^−1^); C-6 (MeCN, 0.1 mg mL^−1^); NiR (MeCN, 0.1 mg mL^−1^).

### Assembly of microreactors

The microreactor consists of two parts: the glass capillary and the 3D printed body. The model of the light-curing printer is Formlabs Form 2 (0.05 mm layer resolution). The cross section of the square channel is 1.5 mm long. The glass capillary tube has an outer diameter of 1 mm and an inner diameter of 580 μm (the nozzle diameter is 200 μm). The capillary tube is bonded to the main structure by an epoxy glue. The inlet of the capillary is connected to a Teflon tube (i-Quip P2790-05). The flow rate is controlled by two syringe pumps (Longer pump LSP01-2A).

### Calculations of energy-transfer efficiency (*Φ*_ET_) and antenna effect^25^

1. Energy-transfer efficiency (Eq. 1)
  1 $$\varPhi_{{{{{\rm{ET}}}}}}=1-\frac{{{F}_{{{{{\rm{DA}}}}}}}}{{{F}_{{{{{\rm{D}}}}}}}}$$
  *F*_D_ and *F*_DA_ are the fluorescence intensity of the donor in the absence and presence of the acceptor, respectively.
2. Antenna effect (Eq. 2)

2 $${{{{{\rm{antenna}}}}}}\,{{{{{\rm{effect}}}}}}=\frac{{I}_{{{{{{\rm{AF}}}}}}\lambda ({{{{{\rm{D}}}}}})}}{{I}_{{{{{{\rm{AF}}}}}}\lambda ({{{{{\rm{A}}}}}})}}$$

*I*_AF*λ*(D)_ is the emission intensity of the acceptor upon donor excitation, and *I*_AF*λ*(A)_ is the emission intensity of the acceptor upon direct excitation.

### DFT calculations

The structural optimization of TPE, C-6, and NiR and the calculation of molecular orbitals are based on Gaussian16. The optimization geometry task is performed using Becke’s three-parameter hybrid functional with the Lee–Yang–Parr correlation functional (B3LYP)^37^ and 6-311G (2*d*, *p*) basis set. Rendering of molecular orbitals and intermolecular interactions using VMD (version 1.9.3) software^38^. Multiwfn 3.8 software is used for wavefunction calculations^39^. The visualization of RDG (reduced density gradient) plots is done using Python 3.9.7 software. Dispersion correction is performed using the Grimme-D3 method^40^.

### Finite-element simulation

Numerical calculations are based on COMSOL Multiphysics software. The initial mixture is generated by a random function such that the mean value of the phase field function is 0 and the standard deviation is 0.05. The Cahn-Hilliard equation (Eq. 3) describes the separation of the two phases:

3 $$\frac{{{\partial }}{\phi }}{{{\partial }}{{{{{\rm{t}}}}}}}{{\nabla }}\cdot \frac{{\gamma }{\lambda }}{{\varepsilon }^{2}}\nabla {\psi }$$

where *Φ* is the dimensionless phase field variable and t is the time. *γ* is the mobility (m^3^ s kg^−1^), *λ* is the mixing energy density (N), and *ε* is the capillary width proportional to the interface thickness (m). *Ψ* is the phase field assistant variable (Eq. 4).

4 $${\psi }=-\nabla \cdot {\varepsilon }^{2}\nabla {\phi }+\left({{\phi }}^{2}-1\right){\phi }$$

*λ* and *ε* are related to the surface tension coefficient through Eq. 5:

5 $$\sigma=\frac{2\sqrt{2}}{3}\frac{{\lambda }}{{{\varepsilon }}}$$

A mapping method is used for mesh profiling. The maximum cell size is 0.028 μm. The dissection results contain 7200 domain cells and 344 boundary elements.

#### Initialization

The random functions (phi_init) is defined as “2 arguments with a mean value of 0.322 and a range of 9 × 10^−5^”. Variables (Vf1_int) is defined as “ intop1(pf.Vf1), mass fraction ”. Integration (intop1) is defined as “ all domain, order of integration: 4 ”. Phase field variable is defined as “ phipf ”. Phase field help variable is defined as “ psi ”. Reciprocal initial interface distance is defined as “ GI ”. Discretization is defined as “ element order is quadratic ”. Initialize phase field variable is defined as “ phi_init(x[1/um],y[1/um]) ”.

#### Boundary conditions

The wetted wall condition is used for all four boundaries of the calculation area. The contact angle is set to 90° Table 2.

**Table 2:** Numerical parameters

Name	Value	Description
pf.epsilon_pf	2.8 × 10^−8^ [m]	Parameter controlling interface thickness
pf.dfdphi	0	phi-derivative of external free energy
pf.sigma	68.41 [mN/m]	Surface tension coefficient (TPE, 1 mg/mL)
pf.sigma	31.39 [mN/m]	TPE, 0.025 mg/mL
pf.sigma	32.75 [mN/m]	Surface tension coefficient (C-6)
pf.mobility	pf.epsilon_pf^2*pf.chi	Mobility
pf.lam	3*pf.sigma*pf.epsilon_pf/sqrt(8)	Mixing energy density
pf.Vf1	1-pf.Vf2	Volume fraction of solute
pf.Vf2	min(1,max(0.5*(1 + phipf),0))	Volume fraction of solvent
pf.phipfReg	min(1,max(phipf,−1))	Regularized phase field variable
pf.chi	5.90 × 10^−4^ [m*s/kg]	Mobility tuning parameter (TPE, 1 mg/mL)
pf.chi	1.02 × 10^−3^ [m*s/kg]	TPE, 0.025 mg/mL
pf.chi	9.50 × 10^−4^ [m*s/kg]	Mobility tuning parameter (C-6)
pf.lref	10*pf.epsilon_pf [m]	Reference length for two phase flow
pf.G0i	nojac(2/pf.lref) [1/m]	Auxiliary wall variable
pf.DwVar	1/GI-1/pf.G0i [m]	Help variable
pf.Dwi	pf.DwVar [m]	Initial interface distance

#### Solver settings

The time-dependent solver performs the direct solution, and the solver is selected as MUMPS.

### Reporting summary

Further information on research design is available in the Nature Research Reporting Summary linked to this article.

## Supplementary information

Supplementary Information

Reporting Summary

## Data Availability

The data for this study are available in this article or are included in the Supplementary Information, which is also available from the corresponding authors upon request. Source data are provided with this paper.

## Source data

Source Data
