# Supplementary material for: Coemissive luminescent nanoparticles combining aggregation-induced emission and quenching dyes prepared in continuous flow
Source: Nat Commun. 2022 Oct 13;13:6034. doi: 10.1038/s41467-022-33857-x (PMC9562343; doi:10.1038/s41467-022-33857-x)
Supplement: Supplementary file 1 — Supplementary Information [file 41467_2022_33857_MOESM1_ESM.pdf]

Supplementary Information

Coemissive Luminescent Nanoparticles Combining Aggregation-induced Emission and Quenching Dyes Prepared in Continuous Flow

C.Li et al.

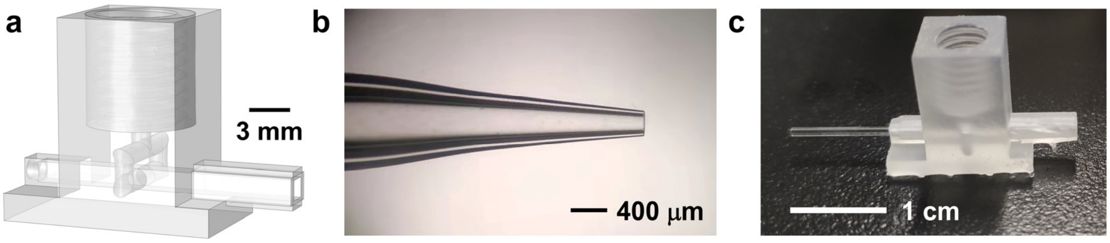

Supplementary Figure 1. a) Model of the microreactor. b) Image of the capillary tube. c) Physical diagram of the microreactor.

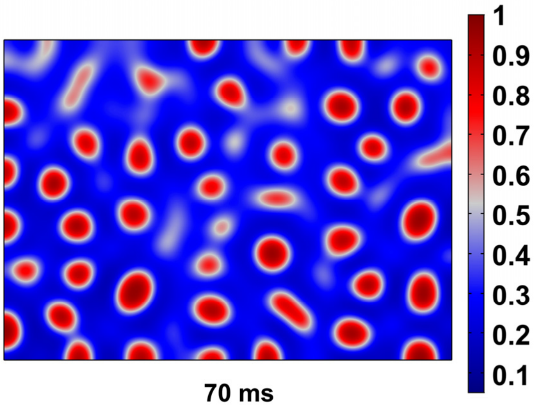

Supplementary Figure 2. Phase field diagram of C-6 at 70 ms.

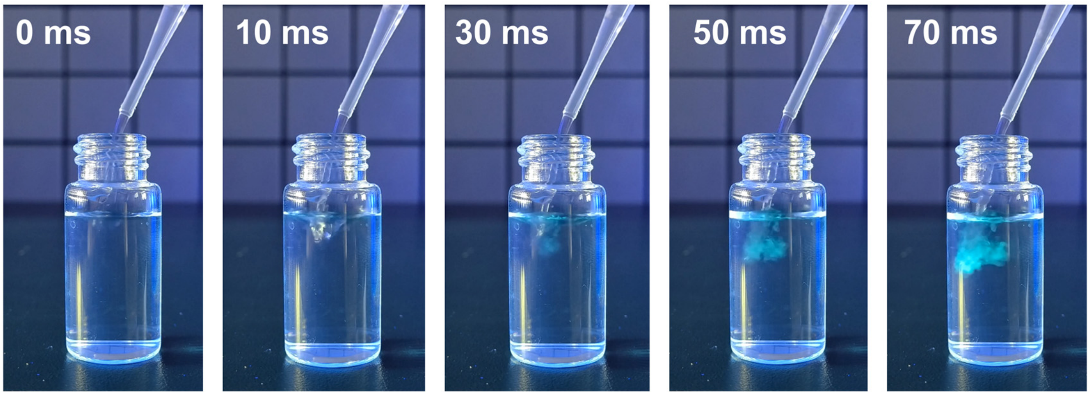

Supplementary Figure 3. Pictures of TPE (1 mg mL^{-1}) precipitation at different moments. Precipitation starts at 50 ms.

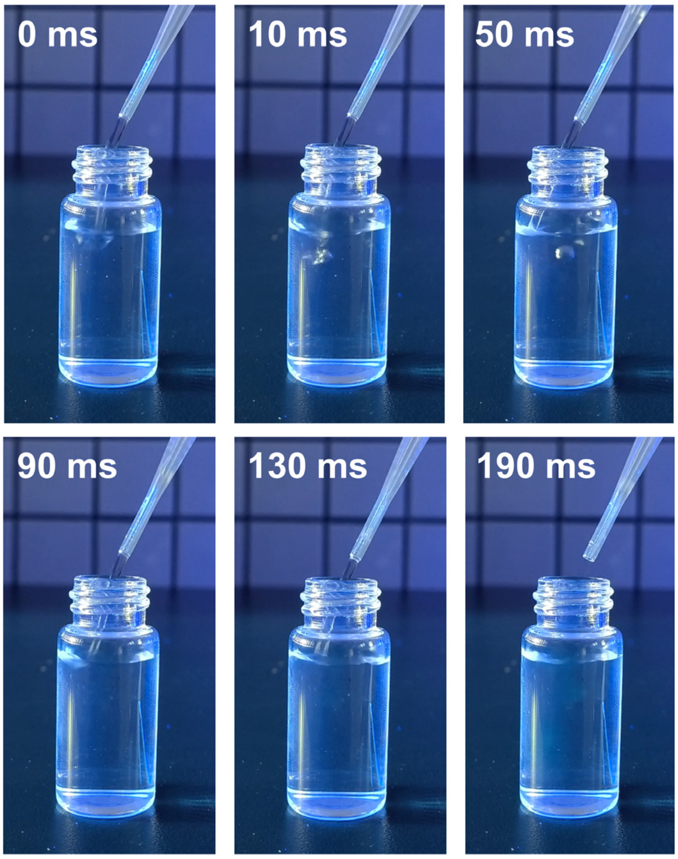

Supplementary Figure 4. Pictures of TPE (0.025 mg mL^{-1}) precipitation at different moments. Precipitation starts at 130 ms.

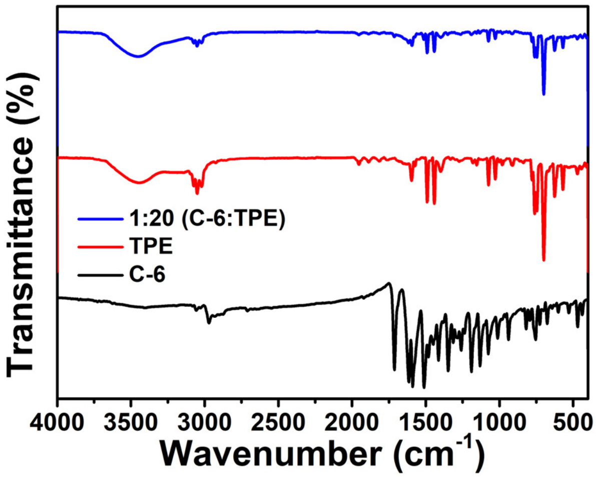

Supplementary Figure 5. IR spectra of TPE, C-6 and C-6@TPE. The two peaks 700 cm^{-1} and 760 cm^{-1} are deformation vibration peaks of monosubstituted benzene rings. The peak at 1600 cm^{-1} is the stretching vibration peak of C=C in the benzene ring. The peak at 3050 cm^{-1} is a stretching vibration peak of C-H in the benzene ring. Source data are provided as a Source Data file.

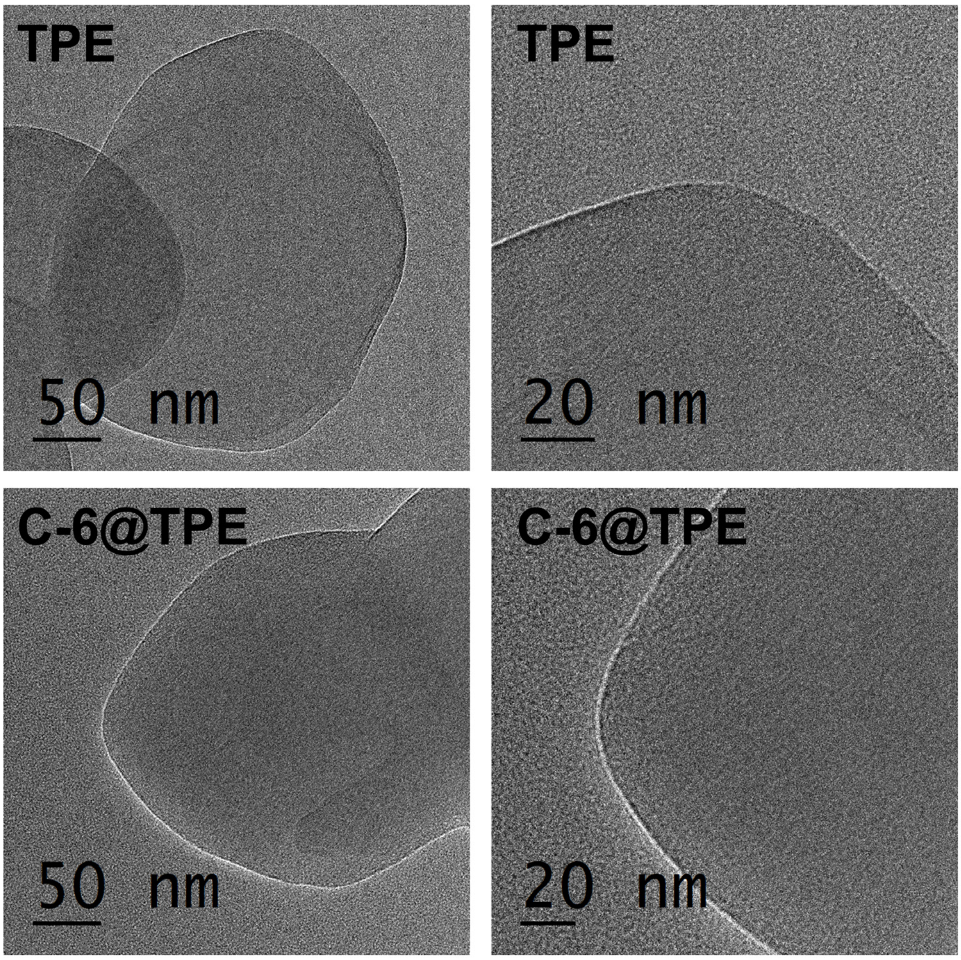

Supplementary Figure 6. TEM images of TPE and C-6@TPE (1:20).

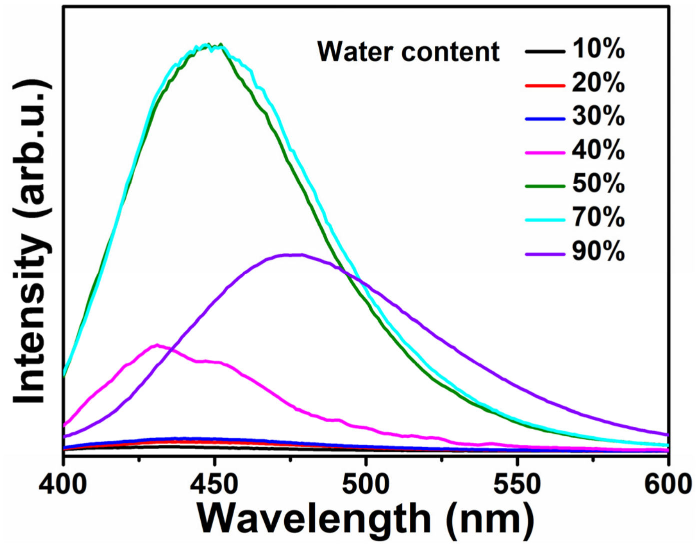

Supplementary Figure 7. Fluorescence spectra of TPE in a solvent mixture of acetonitrile and water. Source data are provided as a Source Data file.

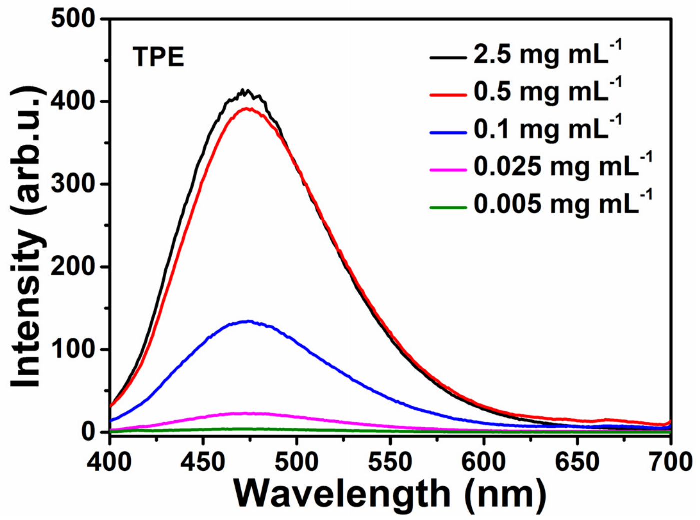

Supplementary Figure 8. Fluorescence spectra of products with different concentrations of TPE precursors. Source data are provided as a Source Data file.

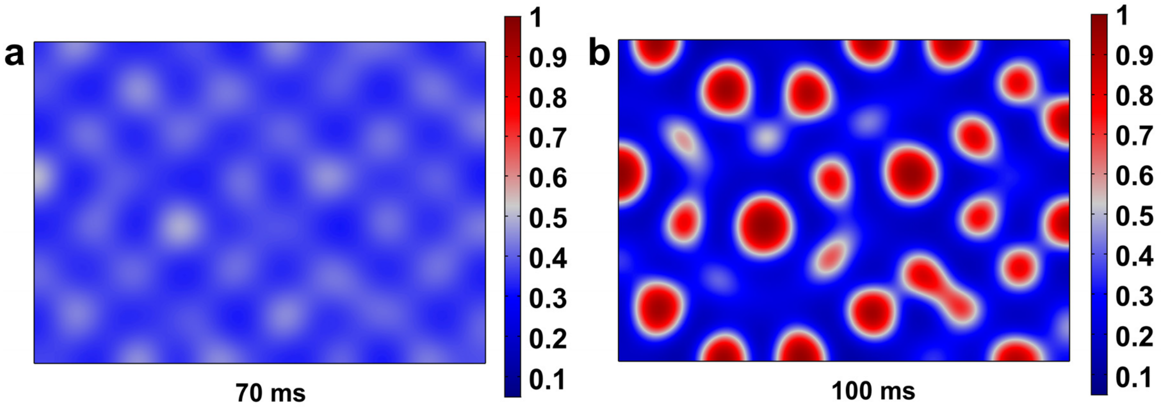

Supplementary Figure 9. a) Phase field diagram of TPE (0.025 mg mL^{-1}) at 70 ms. b) Phase field diagram of TPE (0.025 mg mL^{-1}) at 100 ms.

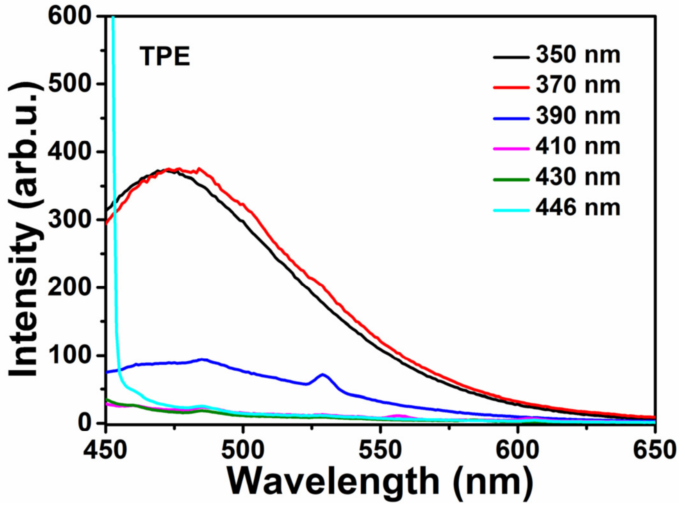

Supplementary Figure 10. Fluorescence spectra of TPE aqueous solutions at different excitation wavelengths. Source data are provided as a Source Data file.

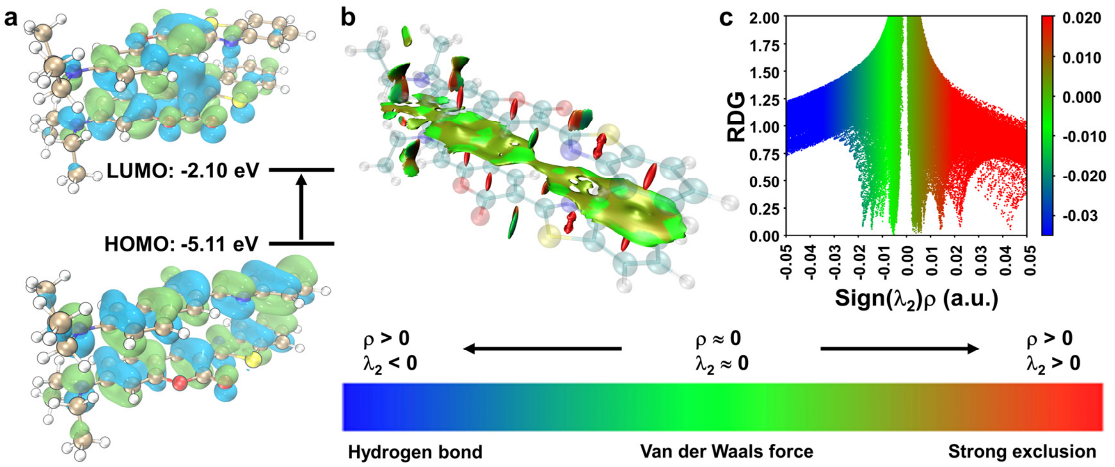

Supplementary Figure 11. a) Aggregation of two C-6 molecules and its orbital energy calculation. b) Noncovalent interactions between C-6 molecules. c) RDG (reduced density gradient) plot between C-6 molecules.

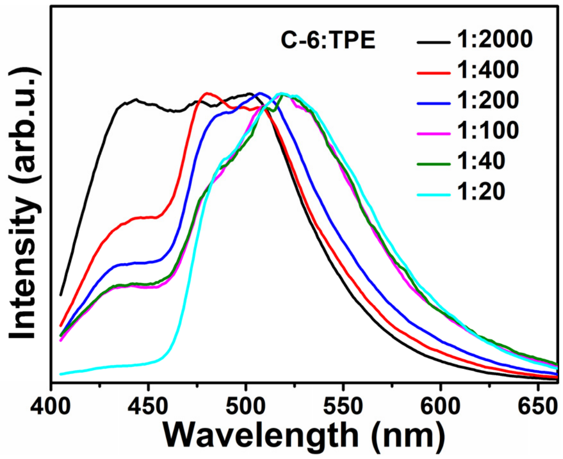

Supplementary Figure 12. Solid-state fluorescence spectra of C-6@TPE at 350 nm excitation light when different ratios of C-6 to TPE were used in the precursors. Source data are provided as a Source Data file.

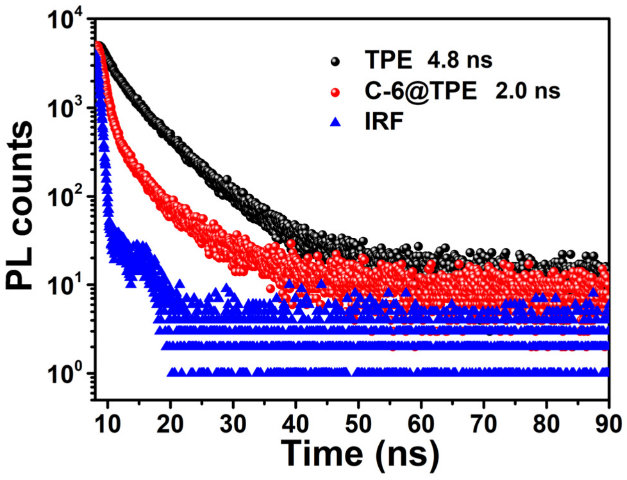

Supplementary Figure 13. Fluorescence lifetime of TPE and TPE in C-6@TPE. Source data are provided as a Source Data file.

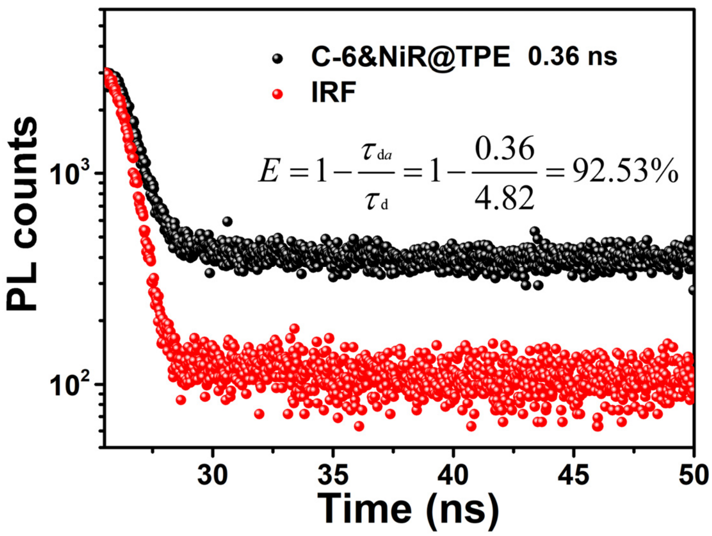

Supplementary Figure 14. Fluorescence lifetime of TPE in C-6&NiR@TPE. Equation for calculating energy-transfer efficiency based on fluorescence lifetime.[1] Where τ_{da} and τ_d are the fluorescence lifetimes of the donor in the presence and absence of the acceptor. Source data are provided as a Source Data file.

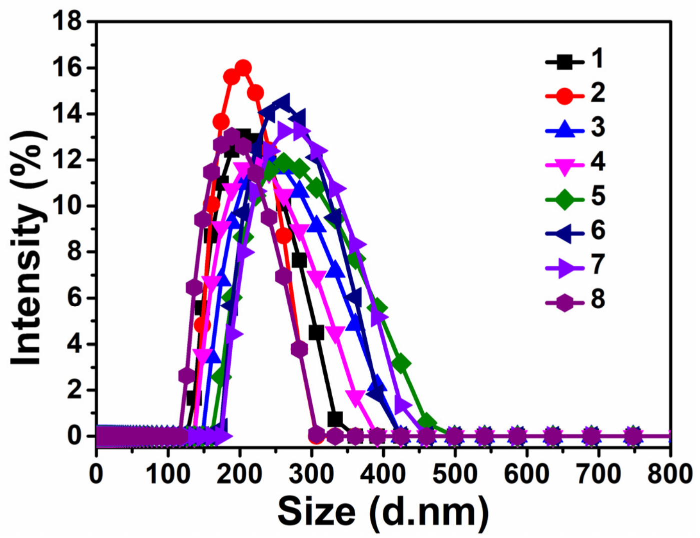

Supplementary Figure 15. Particle size distribution of CEAA dyes with different emission wavelengths (No.1-8). Source data are provided as a Source Data file.

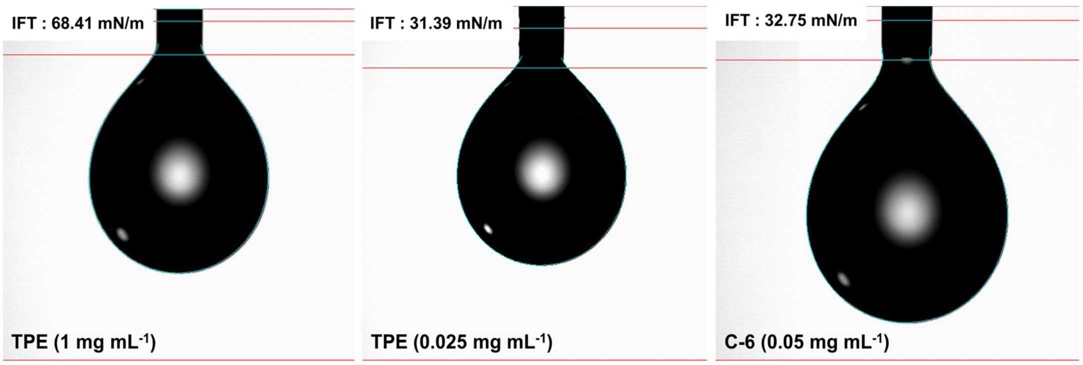

Supplementary Figure 16. Results of surface tension coefficients for different precursor fluids.

Supplementary Table 1. EDS elemental content statistics

Elements	Counts	Mass%
C	68871.53	96.36
O	743.64	2.71
N	3784.71	0.70
S	279.59	0.23

Supplementary Table 2. Fluorescence lifetime fitting results. C-6 in MeCN and C-6 in C-6@TPE. Fluorescence lifetime = Value * (%)

Types of Dyes	τ_1	τ_2	τ_3	χ^2
C-6	2.63 ns (100%)	/	/	1.10
C-6@TPE	0.68 ns (21.87%)	2.91 ns (38.76%)	10.05 ns (39.36%)	1.11

Supplementary Table 3. Fluorescence lifetime fitting results. TPE and TPE in C-6@TPE. Fluorescence lifetime = Value * (%)

Types of Dyes	τ_1	τ_2	τ_3	χ^2
TPE	1.84 ns (34.94%)	6.42 ns (65.06%)	/	1.33
C-6@TPE	0.56 ns (59.28%)	1.90 ns (25.49%)	7.93 ns (15.23%)	1.08

Supplementary Table 4. Fluorescence lifetime fitting results. C-6&NiR@TPE. Fluorescence lifetime = Value * (%)

Types of Dyes	τ_1	τ_2	τ_3	χ^2
C-6&NiR@TPE	0.36 ns (100%)	/	/	1.14

Supplementary references

1. Biju, V., Itoh, T., Baba, Y., and Ishikawa, M. Quenching of Photoluminescence in Conjugates of Quantum Dots and Single-Walled Carbon Nanotube. *J. Phys. Chem. B* **110**, 26068-26074 (2006).
